# Associations between Socioeconomic Status, Social Participation, and Physical Activity in Older People during the COVID-19 Pandemic: A Cross-Sectional Study in a Northern Japanese City

**DOI:** 10.3390/ijerph18041477

**Published:** 2021-02-04

**Authors:** Sachiko Sasaki, Akinori Sato, Yoshie Tanabe, Shinji Matsuoka, Atsuhiro Adachi, Toshiya Kayano, Hiroshi Yamazaki, Yuichi Matsuno, Ann Miyake, Toshihiro Watanabe

**Affiliations:** 1 Department of Physical Therapy, Faculty of Human Sciences, Hokkaido Bunkyo University, Eniwa 061-1449, Japan; a-sato@do-bunkyodai.ac.jp (A.S.); ytanabe@do-bunkyodai.ac.jp (Y.T.); smatuoka@do-bunkyodai.ac.jp (S.M.); 2 Department of Health and Welfare, Long-Term Care Insurance Section, Eniwa City Office, Eniwa 061-1498, Japan; adachi-atsuhiro@city.eniwa.hokkaido.jp (A.A.); kayano-toshiya@city.eniwa.hokkaido.jp (T.K.); yamazaki-hiroshi@city.eniwa.hokkaido.jp (H.Y.); matsuno-yuuichi@city.eniwa.hokkaido.jp (Y.M.); miyake-ann@city.eniwa.hokkaido.jp (A.M.); 3 Department of Health and Nutrition, Faculty of Human Sciences, Hokkaido Bunkyo University, Eniwa 061-1449, Japan; t-watanabe@do-bunkyodai.ac.jp

**Keywords:** coronavirus disease 2019 (COVID-19), socioeconomic status, social participation, motor activity, older adults, community-dwelling

## Abstract

Physical activity (PA) is a key determinant of health in older adults. However, little is known about the effect of social factors on PA among older adults during the coronavirus disease 2019 (COVID-19) pandemic. Therefore, we aimed to clarify the association between socioeconomic status, social participation, and PA during the pandemic. A cross-sectional study was conducted on 999 community-dwelling residents aged 65–90 years. A self-administered questionnaire was used to collect socioeconomic status, social participation, and PA data in August 2020. Multivariable logistic regression analyses were used to calculate the odds ratios (ORs) for the associations between socioeconomic status, social participation, and maintaining PA. For both sexes, PA was reduced by approximately 5–10% after the onset of COVID-19-related distancing restrictions. Men with a low socioeconomic status were less physically active (OR = 0.49, 95% CI: 0.30–0.82). Women who reported social participation had higher odds of maintaining PA (OR = 1.67, 95% CI: 1.13–2.45) during the restrictions. Higher socioeconomic status and social participation levels before the COVID-19 pandemic may have helped older adults to maintain PA during the COVID-19 pandemic. Further research is needed to clarify the potential effects of these factors on the health of older adults.

## 1. Introduction

During the coronavirus disease 2019 (COVID-19) pandemic, national governments have instituted social distancing measures [1]. As a result, approximately three billion people have altered many facets of their everyday life owing to these public health restrictions. While these measures have helped to slow the infection rate, such restrictions have potentially negative health effects. 

Physical activity (PA) is an important determinant of health [2] and is likely to be influenced by social distancing. A global online study performed during the initial stage of the COVID-19 outbreak reported a 27.3% decrease in mean steps [3]. Additionally, recent studies have reported that all PA intensity levels (vigorous, moderate, walking, and overall) have decreased and daily sitting time has increased for people of all ages [3,4]. However, few studies have investigated the changes in PA among older adults during the global pandemic [5,6]. Furthermore, recent data indicate that people who were physically active before the pandemic reported decreased PA due to social restrictions, highlighting the potential importance of maintaining or increasing one’s PA [7]. Additionally, the factors necessary to maintain PA in this age group have not been clarified.

Socioeconomic status affects health and well-being, making health inequalities a major public health concern worldwide [8,9]. Previous studies have shown that those with a high socioeconomic status tend to be the most physically active among older adults. A possible explanation for this is that this group has more leisure time to engage in PA [6,9]. However, the COVID-19 pandemic has resulted in major lifestyle changes, and the effect of socioeconomic status on PA among older adults is not clear.

Social participation is an important contributing factor to active aging [10]. In Japan, 61.0% of the population over 60 years participates in some social activity, and 33.7% participate in social activities related to health or sports [11]. Many studies have suggested that social participation promotes regular PA in older people [12,13]; however, no studies have examined whether greater levels of social participation before the COVID-19 pandemic affected the maintenance of PA in this age group. 

The present study aimed to examine changes in PA levels after the onset of the COVID-19 global pandemic and to clarify the impact of socioeconomic status and social participation on the maintenance of PA among community-dwelling older people in Japan during social distancing restrictions.

## 2. Materials and Methods

### 2.1. Study Design and Population

We conducted a cross-sectional, community-based study in Eniwa, Hokkaido Prefecture, Japan. With reference to previous studies on social participation among Japanese older people [14], we assumed that a 10% difference in PA by social participation could also be obtained in the current study; thus, the required sample size when α = 0.05 and β = 0.10 was 400 individuals who participate in social activities. We used a stratified sampling method: respondents were randomly selected in terms of sex, age (65–74 years and 75–90 years), one of four areas of residence, and social participation before the COVID-19 pandemic, based on the list held by Eniwa City Hall. A total of 2008 enrolled community-dwelling men and women aged 65–90 years were not eligible for elder care. A self-administered questionnaire that inquired about age, sex, height, body weight, self-reported health status, smoking status, alcohol consumption, educational background, family members, socioeconomic status, and social participation was mailed to each participant in August 2020. The questionnaire asked about their current and October 2019 physical activity status. As a result, a total of 27 items were included in the questionnaire. A total of 1493 responders (624 men and 869 women) completed the self-administered questionnaire and returned it by mail (74.4% response rate). Of the 1493 responders, 419 were excluded because of missing physical activity data, and 75 were excluded because of missing other types of data. A total of 999 participants (462 men and 537 women) were finally included in the analysis. This study was conducted according to the Declaration of Helsinki guidelines, approved by the institutional ethics board for epidemiological studies of the Hokkaido Bunkyo University (approval number: 01033), and all participants provided written informed consent. 

### 2.2. Assessment of Physical Activity

The international physical activity questionnaire short form (IPAQ-SF) was used to assess PA [15], as it has shown adequate validity in measuring physical activity and sedentary behavior in older adults in Japan (r  =  0.42–0.53) [16]. The IPAQ-SF consists of seven items and measures the frequency and duration of any walking and moderate-to-vigorous physical activity (MVPA) undertaken for more than 10 continuous minutes during a typical seven-day period. The obtained data were used to estimate the total metabolic equivalents (METs) in minutes per week in accordance with the official IPAQ-SF guidelines. Standardized MET values were assigned for walking (3.3 METs), moderate-intensity activity (4.0 METs), and vigorous-intensity activity (8.0 METs). Additionally, data from the IPAQ-SF were classified into three categories based on the guidelines: low, moderate, and high. We categorized the PA level into two categories: low physical activity (LPA) and MVPA, because current activity guidelines recommend MVPA as a key determinant of health in older adults [3]. Additionally, the total sitting time per week was measured by the question “How much time did you usually spend sitting during a weekday?”

### 2.3. Assessment of Socioeconomic Status

We evaluated the socioeconomic status of participants using their educational level and subjective economic status. Regarding educational level, it is easily recorded and remains stable over an individual’s lifetime [17]. Educational level classification was performed in a previous Asian population-based study [18,19] and categorized as either “high school or less” or “more than high school.” Subjective economic status is an indicator of variances in life, such as income, social integration, or environment [20], and many studies have reported that subjective economic status is associated with health outcomes [21], even when controlling for objective income [22]. We assessed economic status using a subjective measurement of economic status as follows: “How do you feel about your current economic situation?” The assessment of economic status in our study was used in the 2012 Comprehensive Survey of Living Conditions conducted by the Japanese Ministry of Health, Labour, and Welfare [23] and validated by comparing the question with annual household income reported in another study [24]. The five response options were “excellent,” “good,” “normal,” “poor,” and “very poor.” We classified excellent, good, and normal as “normal to good,” and poor and very poor as “poor” [25]. 

### 2.4. Assessment of Social Participation

Social participation was assessed using the list of participants in an exercise program to improve motor function and to prevent nursing care held in community centers for older adults. Previous studies among Asian older populations have shown that those who participate in any social activities at least once a month and continuously participate in social activities have better mental and physical health [26,27]. To focus on regular social participation before the COVID-19 pandemic, we defined “participating in an exercise program” as at least once per month and at least three times per year in each year from 2015 to 2019.

### 2.5. Covariates

Data were collected via a self-administered questionnaire. The following variables were considered as covariates in this analysis: age, sex, body mass index (BMI; calculated as weight (kg)/height (m^2^)), current smoking status (yes or no), current alcohol consumption status (yes or no), self-reported health status (good or poor), and living alone (yes or no). 

### 2.6. Statistical Analysis

The data are presented as the mean ± standard deviation and the percentage (number) of participants in that category. Differences in PA before and after the distancing restrictions were tested using a paired *t*-test or McNemar’s test. To assess the impact of PA maintenance during COVID-19-related restrictions, we divided the participants into two groups based on their level of PA. Participants who had LPA before and after the restrictions and those with MVPA before the restrictions but LPA after were combined into the “decreased activity or maintained LPA” group. Participants who had LPA before the restrictions but MVPA after and those with MVPA both before and after were combined into the “increased activity or maintained MVPA” group. The decreased activity or maintained LPA group was used as the reference category. Multivariable logistic regressions were used to calculate the odds ratios (ORs) and 95% confidence intervals (CIs) for the association between socioeconomic status, social participation, and PA maintenance. The statistical model incorporated the following variables as covariates: age (years as a continuous variable), BMI (kg/m^2^ as a continuous variable), self-reported health status (good or poor), current smoking status (yes, or no), current alcohol consumption (yes or no), and living alone (yes or no). All statistical models were carried out separately by sex. All analyses were conducted using JMP Pro software version 14.0.0 for Macintosh (SAS Institute, Cary, NC, USA). Statistical significance was defined as a two-tailed *p*-value of <0.05.

## 3. Results

### 3.1. Participant Characteristics

The characteristics of the 999 study participants are shown in Table 1. The mean age ± standard deviation was 74.5 ± 6.3 years, and 53.8% were women. Overall, 42 (9.1%) men and 188 (35.0%) women reported social participation (Table 1).

### 3.2. Change in Physical Activity

The changes in PA after the onset of COVID-19 restrictions are shown in Table 2. For both sexes, after the onset of restrictions, PA (expressed as METs in minutes per week) was reduced by approximately 5–10% for moderate-intensity activity, walking, and total PA. After the restrictions, there was an increase in sitting time (5% increase for men, 10% increase for women). Men showed a decrease in high-intensity PA, while women showed no change. Regarding the category of PA, 180 (39.0%) men and 287 (53.5%) women decreased their MVPA or maintained LPA after the restrictions, and 282 (61.0%) men and 250 (46.6%) women increased their PA or maintained their MVPA.

### 3.3. Association between Socioeconomic Status, Social Participation, and Physical Activity

The adjusted odds ratios for increased activity or maintained MVPA by socioeconomic status and social participation are shown in Table 3. Men with a low socioeconomic status were less physically active (OR = 0.49, 95% CI: 0.30–0.82) after adjusting for possible confounding factors, including age, BMI, self-reported health, smoking, alcohol consumption, and living alone. By contrast, women with a low socioeconomic status were not less physically active (OR = 1.03, 95% CI: 0.62–1.60). Women who reported social participation had higher odds of increasing PA or maintaining MVPA (OR = 1.67, 95% CI: 1.13–2.45) after adjusting for possible confounding factors, including age, BMI, self-reported health, smoking status, alcohol consumption, and living alone. Men who reported social participation did not have higher odds of increased activity (OR = 1.48, 95% CI: 0.74–3.00).

## 4. Discussion

This study compared PA among older adults before and after the distancing restrictions due to COVID-19 in a community setting in Japan. After the restrictions, PA levels for both sexes were approximately 5–10% lower, and sitting time increased (by 5% for men and 10% for women). Older men with a lower socioeconomic status were more likely to be less physically active. In contrast, older women with higher social participation were more likely to be physically active during the pandemic. These associations were observed even after controlling for potential confounding factors, including age, BMI, self-reported health, smoking status, alcohol consumption, and living alone.

Several studies have identified a large reduction in PA and increased sedentariness among older people after COVID-related public health restrictions were enacted [5,7]. A recent online study assessed PA using the IPAQ and found that COVID-19 home confinement had a negative effect on all levels of PA and increased the mean sitting time. The study reported a 36.9% decrease in vigorous activity, a 34.7% decrease in moderate activity, a 42.7% decrease in walking, and a 28.6% increase in sitting time [4]. Although the results of this previous study are in accordance with ours, the degree of the decrease in PA and the increase in sedentary time was smaller in our study. While most previous studies have examined the period before and after the declaration of a state of emergency in various countries, we specifically examined changes in PA during the six months following the declaration of a state of emergency in Japan to examine long-term behavioral changes due to the pandemic. This may account for the differences seen in our study compared with previous studies. Because the effects of a large pandemic on people’s behavior and lifestyle are unknown, further research is needed.

Understanding the determinants of PA during the COVID-19 pandemic is crucial for promoting PA and developing public health interventions. PA in older adults is affected by various factors [28]; therefore, it is necessary to focus on social factors in addition to individual biological and psychological factors to understand the reasons for engaging or not engaging in PA [29,30]. Socioeconomic status has a measurable and significant effect on health, such as affecting cardiovascular disease [18], type 2 diabetes [31], and mortality [9]. A recent review found that older people with a higher socioeconomic status are more likely to maintain high levels of PA, while those with a lower socioeconomic status are more likely to remain inactive [28]. In the present study, older men with a lower socioeconomic status were more likely to be less physically active during the pandemic-related social distancing, but this association was not found in women. Gender differences in the association between socioeconomic status and health have been reported [32]. A cross-sectional study among Japanese older adults showed that household income is inversely associated with self-reported health, and that men tend to report worsening health as household income decreases. The findings of these previous studies are consistent with ours. However, further research is needed to clarify the potential importance of socioeconomic status on the health of older adults during the global pandemic. 

Many studies have shown that engaging in social activities has a beneficial effect on health, such as decreasing mortality [8] and coronary heart disease [9] and improving physical function [10] and cognitive function [11]. Social participation is also an important contributing factor for promoting regular PA in older adults [2]. A previous study suggested that the effect of social participation on health differs by sex and that this effect is greater in women than in men [33]. Consistent with the results of previous studies, our results suggest that social participation may contribute to the maintenance of regular PA in older women, even during periods of distancing restrictions. Women are more likely to be socially isolated owing to traditional gender roles such as being responsible for housework [34], which may have emphasized the impact of high social participation on PA. To the best of our knowledge, this is the first study to investigate the association between social participation and PA during the COVID-19 pandemic. Additional studies are needed to confirm these findings. 

A strength of our study is the moderately large sample size and the inclusion of people aged 65–90 years. In addition, we considered BMI, self-reported health status, lifestyle factors, and living alone. These are important determinants of PA, and the remained significant after adjusting for these confounding factors. Additionally, the response rate was acceptable (74.4%). However, this study also had several limitations. First, the PA measure relied on a self-administered questionnaire, which could be susceptible to misclassification, particularly with respect to the level of physical activity before the COVID-19 pandemic. However, the questionnaire we used has been well-established, and many similar epidemiological studies have used it to assess PA. Second, although the response rate was sufficiently high, there was a considerable amount of missing data (33.1%). Compared with responders who thoroughly completed the questionnaire, responders with missing data were more likely to be women, have a low socioeconomic status, and be older. It is possible that participants with a lower economic status were less likely to respond to the questionnaire, so the strength of association might have been underestimated. In addition, while self-reported economic status is a robust indicator of economic capability, it may underestimate economic difficulties among older adults [35]. Third, we could not fully identify the impact of change in PA on our findings, which should be a topic of further research. Fourth, the participants may not necessarily be representative of community-dwelling older adults, especially regarding socioeconomic status. Finally, because this was a cross-sectional study, we were unable to establish the causality of the association between PA and the social participation and socioeconomic status of older adults. 

## 5. Conclusions

Our findings indicate that older men with a lower socioeconomic status are more likely to be less physically active during the pandemic, and older women with higher social participation are more likely to be physically active. These observations have potential implications that could aid the promotion of and recommendations for maintaining PA among individuals of low socioeconomic status. Socioeconomic status is an indicator of older people’s overall life situations; those with a lower socioeconomic status may face greater challenges with health behaviors other than PA and thus may need more social support during a pandemic. Further research is needed to clarify the effects of socioeconomic status and social participation on health during large, acute public health restrictions.

## Figures and Tables

**Table 1:** Demographic and clinical characteristics of the participants in Eniwa, Hokkaido, Japan.

Characteristics	Men	Women
	(*n* = 462)	(*n* = 537)
Age	74.2 ± 6.5	74.7 ± 6.2
Body mass index (kg/m^2^)	23.6 ± 2.8	23.1 ± 3.5
Self-reported health		
Good	392 (84.9)	444 (82.7)
Poor	70 (15.1)	93 (17.3)
Smoking status		
Non-smoker	400 (86.6)	505 (94.0)
Current smoker	62 (13.4)	32 (6.0)
Drinking status		
Non-drinker	178 (38.5)	415 (77.3)
Current drinker	284 (61.5)	122 (22.7)
Living alone		
No	409 (88.5)	417 (77.7)
Yes	53 (11.5)	120 (22.4)
Educational background		
<High school	328 (71.0)	435 (81.0)
≥High school	134 (29.0)	102 (19.0)
Economic status		
Normal to good	381 (82.5)	461 (85.9)
Poor	81 (17.5)	76 (14.1)
Social participation		
No	420 (90.9)	349 (65.0)
Yes	42 (9.1)	188 (35.0)

Variables are presented as mean ± standard deviation or as number (%) of participants in that category.

**Table 2:** Differences in physical activity before and after the onset of COVID-19 restrictions, assessed by self-reported questionnaire.

Variables	Before Restrictions	After Restrictions	Δ ^a^ (Δ%)	*p*-Value
Men				
METs of physical activity (METs·minutes/week)				
Vigorous intensity	1690.6 ± 2668.8	1604.8 ± 2598.2	85.7 (5.1)	0.035
Moderate intensity	1064.7 ± 1332.8	1002.6 ± 1306.4	62.2 (5.8)	0.0024
Walking	922.9 ± 1035.5	877.4 ± 1028.9	45.5 (4.9)	0.0054
Total physical activity	3678.2 ± 4163.1	3484.8 ± 4112.3	193.4 (5.3)	0.0024
Sitting time (minutes/day)	273.4 ± 203.4	287.7 ± 204.1	14.4 (5.3)	<0.001
Physical activity level				
Maintained LPA (%)	159 (34.4)			
Decreased (%)	21 (4.6)			
Maintained MVPA (%)	273 (59.1)			
Increased (%)	9 (1.9)			
Women				
METs of physical activity (METs·minutes/week)				
Vigorous intensity	742.5 ± 1701.3	717.5 ± 1738.0	25.0 (3.4)	0.40
Moderate intensity	712.5 ± 1062.7	644.4 ± 1005.1	68.1 (9.6)	0.0022
Walking	717.2 ± 899.6	647.2 ± 870.5	69.9 (9.7)	<0.001
Total physical activity	2172.1 ± 2873.2	2009.2 ± 2876.6	163.0 (7.5)	<0.001
Sitting time (minutes/day)	243.7 ± 181.5	267.8 ± 191.6	24.1 (9.9)	<0.001
Physical activity level				
Maintained LPA (%)	255 (47.5)			
Decreased (%)	32 (6.0)			
Maintained MVPA (%)	243 (45.2)			
Increased (%)	7 (1.3)			

Variables are presented as the mean ± standard deviation or as number (%). ^a^ The absolute value of the difference in physical activity before and after restriction. Paired *t*-test or McNemar’s test was used to compare each physical activity in each period. Abbreviations: COVID-19, coronavirus disease 2019; METs, metabolic equivalents; LPA, low physical activity; MVPA, moderate-to-vigorous physical activity.

**Table 3:** Odds ratio of increasing or maintaining MVPA for socioeconomic status and social participation in men and women.

Variables	Cases	%	Crude		Model 1		Model 2	
			OR (95% CI)	*p*-Value	OR (95% CI)	*p*-Value	OR (95% CI)	*p*-Value
Men								
Educational background				0.64		0.74		0.85
<High school	328	71.0	1.00		1.00		1.00	
≥High school	134	29.0	1.11 (0.73–1.67)		1.07 (0.71–1.63)		0.96 (0.62–1.48)	
Economic status				<0.001		<0.001		0.0069
Normal to good	381	82.5	1.00		1.00		1.00	
Poor	81	17.5	0.44 (0.27–0.71)		0.42 (0.26–0.69)		0.49 (0.30–0.82)	
Social participation				0.65		0.41		0.27
No	420	90.9	1.00		1.00		1.00	
Yes	42	9.1	1.16 (0.60–2.26)		1.33 (0.67–2.63)		1.48 (0.74–3.00)	
Women								
Educational background				0.91		0.50		0.51
<High school	435	81.0	1.00		1.00		1.00	
≥High school	102	19.0	1.03 (0.67–1.58)		0.86 (0.55–1.34)		0.86 (0.54–1.36)	
Economic status				0.73		0.72		0.90
Normal to good	461	85.9	1.00		1.00		1.00	
Poor	76	14.1	0.92 (0.56–1.50)		0.92 (0.56–1.50)		1.03 (0.62–1.60)	
Social participation				0.09		0.0068		0.0094
No	349	65.0	1.00		1.00		1.00	
Yes	188	35.0	1.37 (0.96–1.95)		1.69 (1.16–2.47)		1.67 (1.13–2.45)	

A logistic regression model was used to calculate the odds ratios. Model 1 was adjusted for age. Model 2 was adjusted for age and body mass index, self-reported health, smoking, alcohol use, and living alone. Abbreviations: MVPA, moderate-to-vigorous physical activity; OR, odds ratio; CI, confidence interval.
